# Alendronate treatment in cats with persistent ionized hypercalcemia: A retrospective cohort study of 20 cases

**DOI:** 10.1111/jvim.16508

**Published:** 2022-10-01

**Authors:** Maxime Kurtz, Loïc Desquilbet, Justine Maire, Fiona Da Riz, Morgane Canonne‐Guibert, Ghita Benchekroun, Christelle Maurey

**Affiliations:** ^1^ Department of Internal Medicine Ecole Nationale Vétérinaire d'Alfort Maisons‐Alfort France; ^2^ Department of Clinical Epidemiology and Biostatistics Ecole Nationale Vétérinaire d'Alfort Maisons‐Alfort France; ^3^ Ecole Nationale Vétérinaire d'Alfort Université Paris Est Créteil, INSERM, IMRB Maisons‐Alfort France

**Keywords:** bisphosphonates, calcium, chronic kidney disease, urolithiasis

## Abstract

**Background:**

Limited information is available concerning treatment of ionized hypercalcemia in cats.

**Hypothesis/Objectives:**

Describe clinical findings in a cohort of cats with persistent ionized hypercalcemia and evaluate long‐term tolerance and efficacy of alendronate in these patients.

**Animals:**

Twenty cats with persistent ionized hypercalcemia of undetermined origin, presented for routine or referral consultation at the teaching hospital of Maisons‐Alfort (France).

**Methods:**

Medical records were retrospectively reviewed. Cats were divided into Group 1 (cats that received alendronate as well as other treatments, n = 11) and Group 2 (cats that did not receive alendronate, n = 9). Survival analysis (Kaplan‐Meier method, log‐rank test, and Cox proportional hazard models) was conducted to compare time to selected outcomes.

**Results:**

Azotemia was present in 15 cats (75%). Alendronate treatment was administered and well tolerated during the entire follow‐up period (median, 9.5 months; interquartile range [IQR], 6.3; 27) in all cats from Group 1, except in 1 cat that developed severe hypophosphatemia, prompting treatment discontinuation. Univariate analysis determined that alendronate treatment was significantly associated with shorter time to reach a 15% decrease in ionized calcium concentration (iCa) from baseline during follow‐up (119 days vs median not reached, *P* = .02). This association was no longer significant after adjustment for age and initial iCa.

**Conclusions and Clinical Importance:**

Alendronate overall was well tolerated with chronic use in this cohort, and can be considered a treatment option for persistent ionized hypercalcemia in cats.

**Abbreviations**
CKD: chronic kidney disease
EDTA: ethylenediamine tetraacetic acid
iCa: serum ionized calcium concentration
IHC: idiopathic hypercalcemia of cats
IRIS: International Renal Interest Society
PTH: parathormone
PUPD: polyuro‐polydipsia
RTHPT: renal tertiary hyperparathyroidism

## 1 INTRODUCTION

Since the early 2000s, the understanding of calcium disorders in cats has progressed considerably. Idiopathic hypercalcemia (IHC) has emerged as 1 of the most common causes of hypercalcemia in cats.^1^ However, information suggests that among cats with IHC, some in fact may have hypercalcemia caused by phosphate‐restricted diets, chronic kidney disease, or both.^2^, ^3^, ^4^ Diagnosis of IHC is made by exclusion. Thus, when faced with an azotemic and hypercalcemic cat, it is particularly difficult to assess whether hypercalcemia is a cause of, or consequence of, the impaired kidney function or the renal support diet.

Historically, treatment of for persistent ionized hypercalcemia has relied on various strategies, including inhibition of intestinal absorption of calcium (using high‐fiber diets^5^ or calcium‐restricted home‐prepared diets^6^), decreasing calcium release from bone using dietary alkalinization and phosphate restriction (eg, by feeding renal support diets^7^, ^8^) or increasing urinary excretion of calcium by increasing dietary sodium chloride,^9^, ^10^ or through the calciuretic effects of furosemide or glucocorticoids.

Although some degree of response to dietary modifications has been reported in cats with persistent hypercalcemia,^5^, ^6^, ^10^, ^11^ results remain highly individual dependent, and efficacy is often transient. To date, veterinary evidence‐based medicine is too scarce to recommend a specific diet over another in the management of IHC.^1^ A recent study reported the successful use of chia seeds to normalize serum calcium concentration for 10 weeks in 3 cats with IHC that failed to respond to high‐moisture industrial pet foods.^12^

Glucocorticoid treatment formerly was advocated for the treatment of IHC and was observed to be efficient (unpublished data).^1^, ^10^ However, approximately one‐third of cats that receive long‐term steroid treatment will develop diabetes mellitus,^13^ and glucocorticoids have been suspected to contribute to the formation of calcium oxalate uroliths by increasing urinary excretion of calcium. Thus, the use of glucocorticoids (or furosemide) usually is reserved for short‐term treatment of severe symptomatic hypercalcemia before another therapeutic modality is implemented or for cases in which other therapeutic options are not possible.^14^

Bisphosphonates have been used in human medicine since the 1970s for the adjunctive treatment of bone cancer and since the 1990s for the treatment of osteoporosis.^15^ Nitrogenous bisphosphonates (pamidronate, alendronate, ibandronate, zoledronate) inhibit osteoclastic activity by altering intracellular protein trafficking and perturbing normal cytoskeleton physiology,^16^ which results in decreased bone resorption and decreased calcium release from bone stores. Reports on the use of bisphosphonates in cats with hypercalcemia are mostly limited to single case descriptions, although the efficacy of PO alendronate recently has been documented in an uncontrolled prospective study involving 12 cats presented with idiopathic hypercalcemia^17^ with a 6‐month follow‐up period. Alendronate administration in cats usually is considered safe and well tolerated,^18^ despite limited data raising the concern of possible toxicity with long‐term administration.^19^, ^20^ Both the efficacy and safety of alendronate in cats with persistent hypercalcemia need to be further evaluated.

Our aims were to describe the clinical and laboratory findings in a cohort of cats with persistent ionized hypercalcemia (presumed to have IHC), to report long‐term outcome and complications associated with alendronate treatment and to investigate whether treatment with alendronate is associated with a decrease in ionized calcium concentration (iCa) in comparison with other (or no) treatments. We hypothesized that alendronate would be safe and associated with a persistent decrease in iCa compared with other (or no) treatments.

## 2 MATERIALS AND METHODS

### 2.1 Case selection and data collection

The medical database at Alfort Veterinary Teaching Hospital (Maisons‐Alfort, France) was retrospectively searched to identify persistent ionized hypercalcemia (serum iCa >1.4 mmol/L on at least 2 serial measurements) in client‐owned adult cats (>9 months) presented for routine or referral consultations between 2008 and 2018.

Cats were included if iCa remained repeatably increased with established causes of hypercalcemia (eg, primary or tertiary hyperparathyroidism, neoplasia, osteolytic processes, granulomatous diseases, vitamin D toxicity) having been excluded or considered unlikely after evaluation by a board‐certified internal medicine specialist. To be considered for inclusion, plasma parathormone (PTH) concentration had to be below or within the lower third of the reference interval. Cats were not included if they had received treatment with glucocorticoids or furosemide within the 30 days before presentation or if medical records were incomplete.

Medical records were reviewed for signalment, current diet, relevant medical history, duration and severity of clinical signs, physical abnormalities, results of blood and urine analysis, diagnostic imaging features (thoracic or abdominal radiographs, abdominal or cervical ultrasonographic examination), final diagnosis, treatment(s) and follow‐up (duration of follow‐up and number of reevaluations).

**FIGURE 1:**
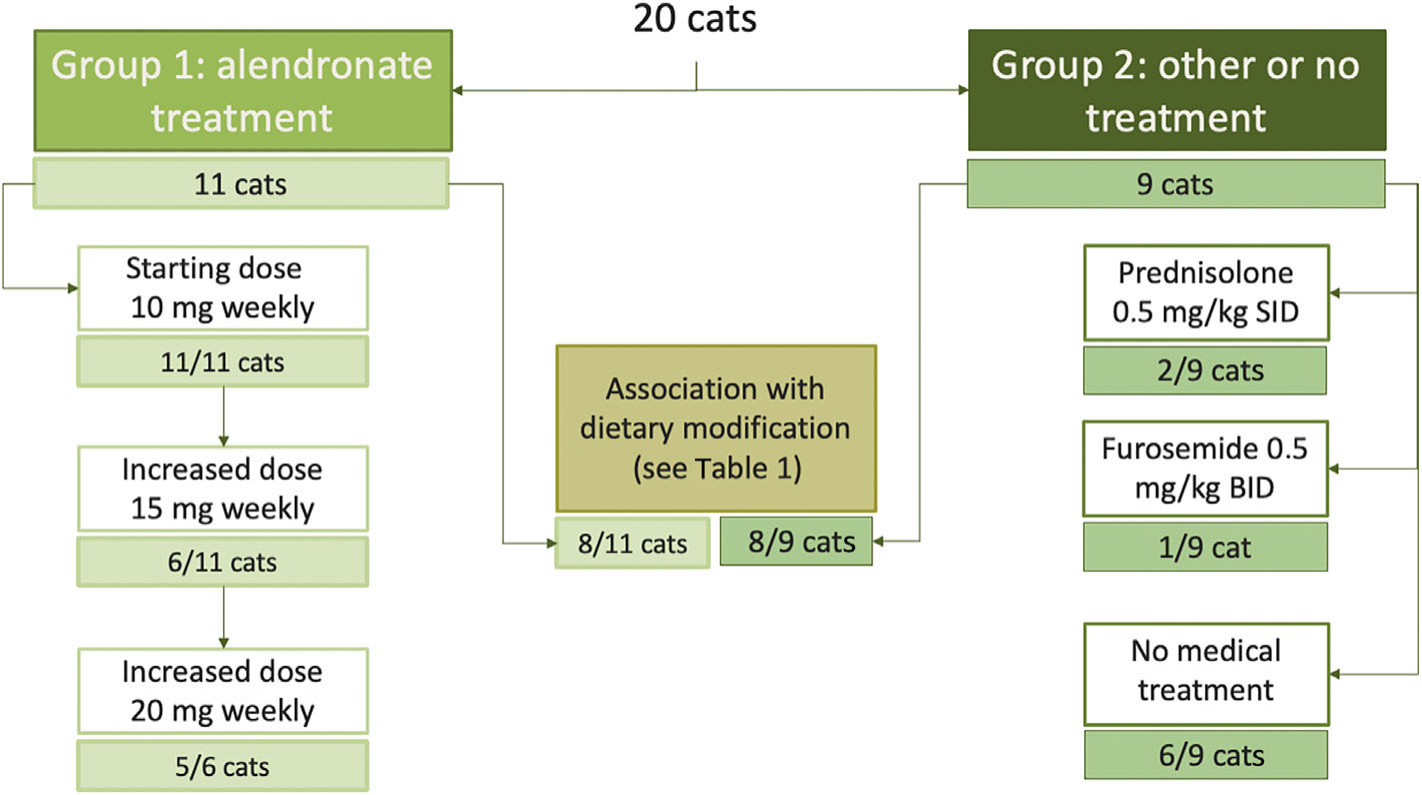
Flowchart indicating group allocation and treatment provision in the studied population

Cats then were divided into Groups 1 (cats treated with alendronate) and 2 (cats that received other or no treatment; Figure 1). Presentation date was defined as the date of first detection of hypercalcemia. The inclusion date (*t*_0_) was defined as the date of the last visit and blood sampling before treatment initiation, and iCa_*t*0_ was defined as iCa at *t*_0_. Two primary outcomes were investigated: the occurrence of normocalcemia (iCa_<1.40_) and the occurrence of a 15% decrease in iCa (iCa_−15%_) in comparison to iCa_*t*0_ during follow‐up. Secondary outcomes included: (1) the percentage of variation in iCa between *t*_0_ and *t*_0_ + 3, 6, 9 and 12 months (±2 months) of follow‐up (defined as [iCa_*t*0+*x* months_ − iCa_*t*0_] × 100/iCa_*t*0_); (2) the percentage of variation in iCa between *t*_0_ and the time of occurrence of the lowest iCa during the follow‐up period ([iCa_min_ − iCa_*t*0_] × 100/iCa_*t*0_); (3) the percentage of episodes of normocalcemia over the total duration of follow‐up; and (4) the percentage of days spent with normocalcemia over the total duration of follow‐up. At the time of a visit (*v*), if iCa was within the reference range, days spent with normocalcemia were calculated as the number of days between visits *v* and *v* − 1.

### 2.2 Analytical procedures

Serum iCa was measured using an ion‐specific electrode analyzer (Nova CRT8, Biomedical, Mississauga, ON, Canada) that previously has been validated in cats.^21^ Blood was collected anaerobically into separator gel tubes, and samples were refrigerated and analyzed within 2 hours after collection. For the measurement of plasma PTH concentration, blood was collected into an EDTA‐coated tube and immediately centrifuged at 4°C, and plasma then was stored at −20°C for 24 to 48 hours until shipment to the laboratory. Serum iCa was determined at the same time as PTH. Intact (1‐84) PTH was measured by ELISA (Immutopics International, San Clemente, California).

Azotemia was defined as a serum creatinine concentration >1.78 mg/dL. Chronic kidney disease (CKD) was diagnosed if cats presented with azotemia combined with decreased urine specific gravity (USG < 1.035) or if azotemia was detected on separate analyses at least 2 weeks apart.

### 2.3 Treatment and follow‐up

When implemented, alendronate treatment was started at an initial dose of 10 mg per cat PO once weekly (Teva Santé, Paris, France). Owners were instructed to fast the cat for 12 hours before and 6 hours after administration and to give the pill PO with water or butter to prevent retention in the esophagus. All cats that did not receive alendronate were evaluated before the publication of a prospective study reporting the use of bisphosphonates in IHC.^17^ The timing of reevaluations was left to the discretion of the clinician. The end of the follow‐up period was defined either as the last reevaluation before the cat was lost to follow‐up or the last reevaluation before the end of the study period.

### 2.4 Statistical analysis

All statistical analyses were performed using commercially available software (SAS University Edition). Continuous variables are presented as medians with their interquartile range (IQR) and were compared using the Mann‐Whitney *U* test. Survival analysis (Kaplan‐Meier method, log‐rank test, and Cox proportional hazard models) was used to compare time to outcomes (iCa_<1.40_ and iCa_−15%_) between cats receiving alendronate (Group 1) and cats receiving either another treatment or no treatment (Group 2) at *t*_0_. Survival time was defined as the time between *t*_0_ and the outcome date or the censoring date. Censored cats were cats lost to follow‐up and cats that did not manifest the outcome before the end of the study. Censoring date was defined as the last reevaluation for which the outcome had not been observed. Crude and adjusted hazard ratios (HRs) along with their 95% confidence intervals (95% CIs) were calculated using Cox proportional hazards models. The variables included in the multivariate model as potential confounders were age and iCa_*t*0_. Statistical significance was set at *P* < .05.

## 3 RESULTS

### 3.1 Clinical findings

Twenty cats were included. Group 1 (n = 11) included 4 Birman cats, 3 Persians, 1 Norwegian and 3 domestic shorthair cats, which were included between 2016 and 2018. Six cats were neutered males, and 5 cats were spayed females. Group 2 (n = 9) included 1 Chartreux, 1 Exotic Shorthair, 1 Persian, 1 British Shorthair and 5 domestic shorthair cats, which were included between 2008 and 2016. Six cats were neutered males, and 3 cats were spayed females. The median age at presentation was 8 years (IQR, 7; 11.5) in Group 1 and 16 years (IQR, 9; 18) in Group 2. Ten cats (50%) were longhaired. Ten cats had been diagnosed previously with CKD: 4 in Group 1 (36%, all 4 International Renal Interest Society [IRIS] stage 2), and 6 in Group 2 (67%, IRIS stages 2 [n = 2], 3 [n = 3], and 4 [n = 1]). The median time between the detection of CKD and presentation was 1131 days (IQR, 789; 1270) in Group 1 and 178 days (IQR, 32; 394) in Group 2.

Eleven (55%) and 3 (15%) cats were fed a renal support diet and an acidifying urolithiasis management diet, respectively, at presentation (Table 1). Two cats (10%) had received calcium‐containing phosphate binders before presentation. The time between initiation of renal support or acidifying diet and presentation was not reported in most cats.

**TABLE 1:** Repartition of the type of diet fed before and after treatment initiation (*t*_0_) among groups

Group	n	Pretreatment diet	n	Dietary modification
1	6	Renal support diet	3	Fiber supplementation (psyllium)
	3	Urolithiasis management diet	2	Renal support diet
			1	Fiber supplementation (psyllium)
	2	Physiological	1	Renal support diet
			1	Fiber‐rich diet
2	5	Renal support diet	3	Fiber supplementation (psyllium)
			1	Fiber‐rich diet
	2	Physiological	2	Renal support diet
	2	Unknown	1	Renal support diet
			1	Fiber‐rich diet

*Note*: High‐fiber diet: Fiber Response diet, Royal Canin, Aimargues, France. Psyllium supplementation: Fiberact, MP Labo, Grasse, France.

The most common clinical signs were vomiting (n = 8, 40%), weight loss (n = 7, 35%) and polyuria and polydipsia (PUPD; n = 6, 30%). Other chief complaints are presented in Table 2. Five cats (25%) did not have any clinical signs and were presented for the reevaluation of subclinical CKD or for geriatric evaluation.

**TABLE 2:** Clinical signs documented at presentation in the studied population

Clinical signs	Number of cats	Percentage
Vomiting	8	40
Weight loss	7	35
Polyuria‐polydipsia	6	30
Dysorexia	3	15
Constipation	3	15
Pollakiuria	3	15
Hematuria	3	15
Periuria	2	10
Lethargy	1	5
None	5	25

### 3.2 Clinicopathological findings

At presentation, median serum creatinine concentration was 1.8 mg/dL (IQR, 1.06; 2.30), and median serum phosphorus concentration was 4.6 mg/dL (reference range, 3.2‐7.8). Azotemia was present in 6 cats in Group 1 (55%, all of which were classified as IRIS stage 2) and in 9 cats in Group 2 (100%; 7/9 classified as IRIS stage 2 and 1 each classified as stage 3 and 4, respectively). All cats with reported PUPD were azotemic. Low, normal, and high serum phosphorus concentrations were observed in 1, 18, and 1 cats, respectively. Serum phosphorus concentrations exceeded IRIS recommended targets for cats with CKD in 4 cats in Group 1 and in 5 cats in Group 2. Biochemical and urinalysis variables for both groups are presented in Table 3.

**TABLE 3:** Clinicopathological data at presentation in the studied population

Parameter		Ionized calcium	Total calcium	Urea	Creatinine	Phosphorus	PTH	USG
Reference range		1.1‐1.4	8.0‐10.5	40‐80	0.52‐1.78	3.2‐7.8	50‐200	
Unit		mmol/L	mg/dL	mg/dL	mg/dL	mg/dL	pg/mL	
Median (IQR)	Group 1	1.62 [1.53; 1.7]	12.6 [12.4; 12.6]	64 [53; 78]	1.8 [1.06; 2.3]	4.55 [3.75; 4.78]	32 [20; 51]	1.028 [1.020; 1.036]
	Group 2	1.53 [1.49; 1.58]	12.65 [11.65; 13.2]	103 [71; 120]	2.2 [2.0; 2.6]	4.6 [4.5; 4.7]	20 [20; 41]	1.027 [1.020; 1.034]
n	Group 1	11	5	11	11	10	11	11
	Group 2	9	6	9	9	9	9	8
No. increased	Group 1	11	5	3	6	0	0	–
	Group 2	9	5	9	9	1	0	–
No. decreased	Group 1	0	0	0	0	1	8	–
	Group 2	0	0	0	0	0	7	–

Abbreviations: IQR, interquartile range; PTH, parathormone; USG, urine specific gravity.

Eleven cats had iCa and serum total calcium concentration measured on the same sample at presentation, and 10 of them (91%) had serum total hypercalcemia (median serum total calcium concentration of 12.6 mg/dL [IQR, 12.2; 13.1]; reference range, 8.0‐10.5 mg/L). Median plasma PTH concentration was 32 pg/mL (IQR, 20; 51) in Group 1 and 20 pg/mL (IQR, 20; 41) in Group 2 (reference interval, 5‐200 pg/mL). Plasma PTH was undetectable in 10 of 20 cats (50%), detectable but below the lower end of the reference interval in 5 of 20 cats (25%) and within the lower third of the reference interval in 5 of 20 cats (25%).

Median iCa at presentation was 1.62 mmol/L (IQR, 1.53; 1.70) in Group 1 and 1.53 mmol/L (IQR, 1.49; 1.58) in Group 2.

### 3.3 Medical imaging

Abdominal ultrasonography was performed at presentation in 19 cats (95%). Upper, lower, and both upper and lower urinary tract lithiasis was diagnosed in 4 (20%), 2 (10%), and 2 (10%) cats, respectively. Four cats (20%) had renal parenchymal mineralization. Abdominal radiographs were evaluated in 9 cats (45%), and radiopaque uroliths were identified in the upper (n = 4) or lower (n = 1) urinary tract or both (n = 1).

### 3.4 Treatment modalities

Treatment modalities are presented in Figure 1. All cats in Group 1 received an initial dose of 10 mg of alendronate PO weekly. The median time between presentation and *t*_0_ was 60 days (IQR, 12; 125). Six cats had their dose increased to 15 mg weekly because of an insufficient decrease in iCa, and the median time between *t*_0_ and first dose escalation for these 6 cats was 42 days (IQR, 27; 55). Five of these 6 cats had their weekly dose further increased to 20 mg after a median of 92 days (IQR, 28; 193) from the first increase. Alendronate was administered throughout the duration of the follow‐up period in 10/11 cats from Group 1 and was discontinued in 1 cat because of hypophosphatemia (see follow‐up and outcome).

One cat in Group 2 did not receive any medical treatment or dietary modification. Three cats in Group 2 (33%) received medical treatment, which included prednisolone in 2 cats (22%) and furosemide in 1 cat. Prednisolone was given PO at a dosage of 0.5 mg/kg/day for 3 months (1 cat) and 2 months (1 cat), followed by tapering the dosage to 0.5 mg/kg q48h and progressive treatment withdrawal. Prednisolone then was administered during periods when clinical signs attributable to hypercalcemia (eg, dysorexia, vomiting) were noted. Furosemide was given PO at a dosage of 0.3 mg/kg q12h for 15 days.

Eight cats in Group 1 (73%) and 8 cats in Group 2 (89%) underwent dietary modification from *t*_0_, as described in Table 1. No dietary change was further reported in any cat after *t*_0_. Phosphate binders were not discontinued during the follow‐up period in either of the 2 cats that were treated at *t*_0_. Among cats that had their food changed to a renal support diet, 1/3 (Group 1) and 3/3 (Group 2) were azotemic at the time of food change.

### 3.5 Follow‐up and outcome

The median duration between *t*_0_ and the last reevaluation was 9.5 months (IQR, 6.3; 27) in Group 1 and 15 months (IQR, 9; 18.5) in Group 2. The median number of check‐up visits was 6 (IQR, 2.5; 10) in Group 1 and 4 (IQR, 3; 6) in Group 2.

**FIGURE 2:**
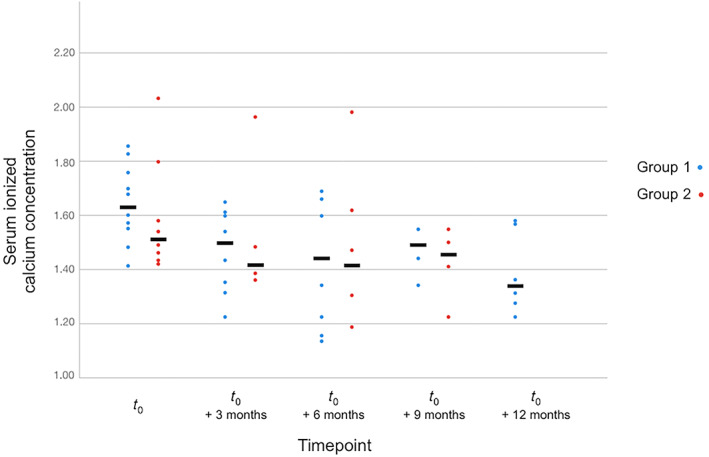
Scatter plot of serum ionized calcium concentration at each follow‐up timepoint (±2 months) between cats from Group 1 and Group 2

**FIGURE 3:**
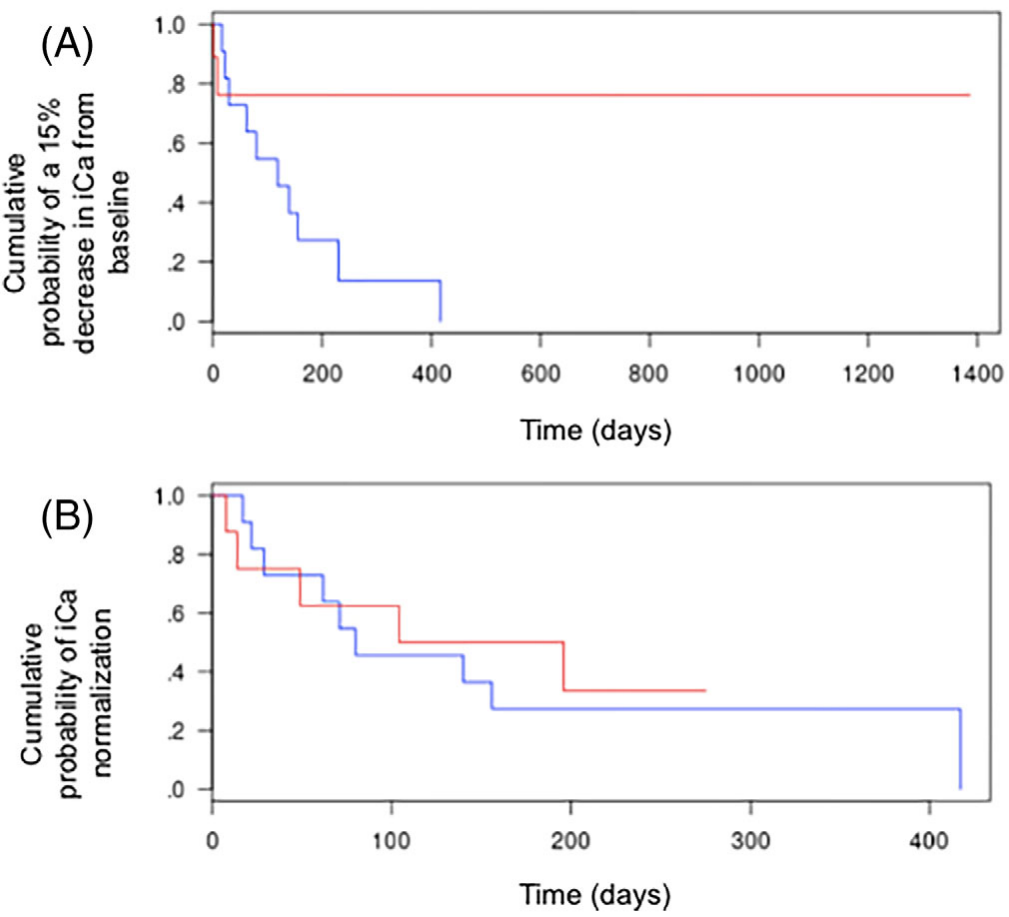
Kaplan‐Meier estimates for the occurrence of a 15% decrease in ionized calcium concentration (iCa) from baseline (A) and occurrence of normocalcemia (B) between baseline and the last check‐up. The median time to a 15% decrease in iCa was significantly different in Group 1 compared with Group 2 (*P* = .02). No significant difference was noted when evaluating the time to occurrence of normocalcemia (*P* = .81). Blue curve: treatment with alendronate (Group 1); red curve: other or no treatment (Group 2)

Figure 2 presents iCa results at each timepoint for the 2 groups. The median time to iCa_−15%_ was significantly shorter in Group 1 (119 days) than in Group 2 (median not reached; *P* = .02; Figure 3A). The corresponding crude HR was 5.0 (95% CI, 1.1‐23.5; *P* = .04). After adjusting for age and iCa_*t*0_, alendronate treatment was no longer significantly associated with a shorter time to iCa_−15%_ (HR, 3.8; 95% CI, 0.6‐24.4; *P* = .16). Median times to iCa_<1.40_ were not significantly different between Group 1 (80 days) and Group 2 (150 days; *P* = .81; Figure 3B). The corresponding crude HR was 1.5 (95% CI, 0.4‐3.5; *P* = .81). This result remained unchanged after adjustment for age and iCa_*t*0_. Secondary outcomes were not significantly different between the 2 groups (Table 4). One of 11 (Group 1) and 6/9 (Group 2) cats were lost to follow‐up during the study period because further evaluations were made by the referring veterinarians.

**TABLE 4:** Secondary outcomes at each time point in both groups

Parameter		Group 1	Group 2	
% of variation in iCa at 3 months (±2 months)	Median	−12.3	−0.7	*P* = .37
	IQR	[−19.5; −2.8]	[−7.4; 3.5]	
	n	10	5	
% of variation in iCa at 6 months (±2 months)	Median	−17.8	−1.3	*P* = .35
	IQR	[−21.3; 3.2]	[−6.2; 3.3]	
	n	9	7	
% of variation in iCa at 9 months (±2 months)	Median	−5.0	−1.0	*P* = .34
	IQR	[−6.9; −5.0]	[−11.1; 1.8]	
	n	4	4	
% of variation in iCa at 12 months (±2 months)	Median	−13.8		
	IQR	[−15.2; −6.1]		
	n	6	0	
% of variation in iCa between iCa_*t*0_ and minimal iCa	Median	−23.7	−7.4	*P* = .08
	IQR	[−27.2; 19.9]	[−13.9; −2.0]	
	n	11	9	
% of episodes of normocalcemia over the total number of follow‐up	Median	50.0	25.0	*P* = .20
	IQR	[21.8; 54.6]	[0; 50.0]	
	n	11	9	
% of days spent with normocalcemia over the total duration of follow‐up	Median	32.9	8.7	*P* = .11
	IQR	[10.7; 41.6]	[0; 20.2]	
	n	11	9	

*Note*: Entries where data are missing have been left blank. Group 1 refers to cats treated with alendronate, and Group 2 refers to cats that received other or no treatment.

Abbreviations: IQR, interquartile range; iCa, ionized calcium concentration.

Treatment tolerance was estimated to be good in most cats. At the last reevaluation, the medians of serum phosphorus concentrations were similar between Group 1 (4.8 mg/dL [IQR, 3.2; 5.0]) and Group 2 (5.0 mg/dL [IQR, 4.0; 6.2]; reference interval, 3.2‐7.8 mg/L; *P* = .36). One cat developed severe hypophosphatemia (1.4 mg/dL) 292 days after *t*_0_ and 167 days after having had its dose of alendronate increased to 20 mg weekly. Ionized serum calcium concentration was 1.39 mmol/L at that time (reference interval, 1.10‐1.40). No clinical findings attributable to hypophosphatemia were noted (eg, weakness, hemolytic anemia). Alendronate treatment was discontinued; serum phosphate and ionized calcium concentration increased to 2.9 mg/dL and 1.69 mmol/L, respectively at reevaluation 1 month later. In 2 cats, mild, spontaneously‐resolving lethargy and inappetence were reported during the days after alendronate administration. No other signs of toxicity, such as regurgitation or lameness, were observed. Medians of variation of serum creatinine concentration at the end of the follow‐up period compared to serum creatinine concentration at *t*_0_ were similar between Group 1 (8% [IQR, −9; 44]) and Group 2 (7% [IQR, −6; 34]; *P* = .86). Three of 11 cats in Group 1 and 2/9 cats in Group 2 experienced significant increases in serum creatinine concentration (defined as a 30% increase in serum creatinine concentration without an identifiable prerenal cause) during the follow‐up period. Abdominal ultrasonographic examination eliminated ureteral obstruction as a cause for progressive azotemia in 2 cats from Group 1 but not in the others. Clinicopathological data at the end of each cat's follow‐up period are presented in Table 5.

**TABLE 5:** Clinicopathological data at the end of each cat's follow‐up period in the study population

Parameter		Ionized calcium	Creatinine	Phosphorus
Reference range		1.1‐1.4	0.52‐1.78	3.2‐7.8
Unit		mmol/L	mg/dL	mg/dL
Median (IQR)	Group 1	1.32 [1.27; 1.47]	19 [14; 27]	4.8 [3.2; 5.0]
	Group 2	1.50 [1.34; 1.55]	29 [23; 38]	5.0 [4.0; 6.2]
n	Group 1	9	11	9
	Group 2	11	7	8
No. increased	Group 1	4	6	0
	Group 2	6	7	0
No. decreased	Group 1	0	0	1
	Group 2	0	0	1

Abbreviation: IQR, interquartile range.

## 4 DISCUSSION

Our study is the first to report a cohort of cats with persistent ionized hypercalcemia treated with alendronate and followed for a long period of time. We initially aimed at evaluating alendronate in IHC. However, diagnosis of IHC is a process of exclusion and most often requires the analysis of phosphorus and calcium metabolic hormones, including PTH, PTH‐related peptide (PTHrp), 25‐hydroxyvitamin D and 1,25‐dihydroxyvitamin D, to eliminate primary or tertiary hyperparathyroidism, malignancy‐associated hypercalcemia, or vitamin D toxicity.^22^, ^23^, ^24^ In this context, a major difficulty consists of eliminating a renal origin for hypercalcemia. Renal disease itself generally is mentioned as a cause for total hypercalcemia rather than ionized hypercalcemia,^25^ but ionized hypercalcemia can occur with concurrent CKD in cats. Underlying mechanisms include renal tertiary hyperparathyroidism (RTHPT) and phosphate restriction‐related hypercalcemia. Renal tertiary hyperparathyroidism is characterized by the clonal expansion of parathyroid cells that have an altered set‐point for their calcium‐sensing ability and that have become autonomous under the influence of longstanding secondary hyperparathyroidism, most frequently in advanced CKD.^26^ However, RTHPT has not been convincingly identified in cats. Moreover, humans with RTHPT most frequently have severely increased serum phosphate and PTH concentrations,^27^, ^28^ which was not the case in any of our cats: 75% of them had undetectable PTH concentrations, which is similar to what typically is reported in IHC and excludes RTHPT. The remainder did, however, have PTH concentrations within the lower third of the reference range, which also has been reported in IHC and might be attributable to incomplete inhibition of PTH secretion despite increased serum calcium concentrations.^29^ Phosphate‐restricted diets recently have been mentioned as possible factors in the pathogenesis of hypercalcemia in cats with CKD.^2^, ^3^ Eleven cats (55%) in our cohort were fed a phosphate‐restricted renal support diet at the time hypercalcemia was detected. For this reason, some cats in our cohort may have had food‐associated ionized hypercalcemia. Diet was not changed during the study period, which further hindered our understanding of the effects of diet on iCa. Other causes of ionized hypercalcemia in cats include vitamin D toxicity, paraneoplastic disease, severe and diffuse osteolytic processes, granulomatous diseases, and, less frequently, hypoadrenocorticism, hypervitaminosis A, and thyrotoxicosis.^24^ All of these diseases were either excluded or judged unlikely in our cohort given the extensive initial diagnostic evaluation and follow‐up course. To summarize, it is likely that a considerable proportion of cats in our cohort had IHC, but other causes (phosphate‐restricted food, CKD or both) cannot be ruled out. Thus, it is prudent to avoid use of the word “idiopathic” in this context. In the future, this term should be reserved to situations where CKD or diet is definitively excluded as a cause for hypercalcemia.

As stated above, azotemia was a frequent finding in our cohort. Fifty percent of cats had a history of CKD, and 75% were azotemic at the time of first detection of hypercalcemia. Although many sources mention progressive decline in kidney function as a consequence of chronic hypercalcemia, few studies report the prevalence of CKD at the time of detection of hypercalcemia in cats. Thus, understanding of interactions between the kidney and calcium homeostasis in cats remains incomplete. In a case series of 12 cats, only 2 (17%) were mildly azotemic (serum creatinine concentrations of 2.1 and 3.1 mg/dL) at the time of detection of IHC.^17^ Despite the fact that the age of cats was not reported in the aforementioned study, we believe the high proportion of CKD in our cohort may be related to the inclusion of geriatric cats (median age in Group 2, 16 years old). Moreover, it is possible that azotemic cats are overrepresented in our cohort because some of the cats were diagnosed with hypercalcemia during monitoring of renal disease, because iCa evaluation is part of routine blood analyses for patients with CKD at our institution.^28^, ^29^

Chronic longstanding hypercalcemia is associated with insidious and progressive deterioration of quality of life because of weight loss, decreased appetite, upper or lower urinary tract calcium oxalate urolithiasis, ureteral obstruction and progressive decline in kidney function.^22^, ^23^, ^30^ Similar to our study, clinical signs in affected cats are typically vague and nonspecific. Severe clinical signs usually are detected when iCa exceeds 1.9 mmol/L.^1^ A history of PUPD was reported in 1 of 3 cats in our study, which is higher than the rate reported in previously published studies. The frequent PUPD in our cohort is more likely attributable to CKD than to hypercalcemia, because cats seem more resistant to the effects of calcium on urine concentrating ability than dogs. Indeed, in dogs, PUPD is reported in up to 90% of hypercalcemic patients,^31^ presumably because of a combination of decreased renal tubular sodium reabsorption and impaired action of antidiuretic hormone. Conversely, in a series of cats with serum total hypercalcemia, low urine specific gravity was observed almost exclusively in cats with concurrent CKD.^17^, ^22^

We have observed an increasing prevalence in cats of nephrolithiasis‐associated CKD in recent decades. The true prevalence and implications of calcium metabolism disorders in cats with upper urinary tract disease need to be further investigated. Hypercalcemia‐associated urinary tract lesions include renal and ureteral lithiasis, renal parenchymal mineralization, and calcium‐induced nephrosclerosis. Upper urinary tract lithiasis is frequent in IHC and has been reported in 15% to 35% of affected cats,^23^, ^32^ with calcium oxalate uroliths being confirmed in 66% of these cats. Accordingly, upper urinary tract lithiasis was identified on ultrasonographic examination in 6 of 19 cats (32%) in our cohort. Abdominal radiographs identified radiopaque upper urinary tract calculi consistent with calcium oxalate urolithiasis in 5 of 9 cats (55%). The high prevalence of urinary lithiasis in our cohort emphasizes recent data identifying chronic hypercalcemia as a possible risk factor for calcium oxalate lithiasis in cats.^33^, ^34^

Ours is the first study comparing the use of alendronate with other therapeutic modalities in cats with persistent hypercalcemia. Administration of alendronate was significantly associated with shorter time to a 15% decrease in iCa compared with other or no treatments. This association however was not significant after adjustment for age and initial iCa. The threshold of 15% was chosen because iCa exhibits spontaneous variation in cats with IHC^1^ (possibly up to 10%) and because, in a former study, median changes in iCa compared with baseline were − 13.2%, −15.9%, and − 18% after 1, 3, and 6 months of treatment, respectively.^17^ Other evaluated outcomes (Table 3) were not significantly different between the 2 groups.

The overall tolerance of alendronate was estimated to be good in our study and in others.^17^, ^35^ Two of 11 cats showed mild, self‐resolving inappetence after alendronate administration. Gastrointestinal upset is a common adverse effect in humans receiving alendronate and is probably related to the acidic nature of the active drug.^36^ Esophagitis and, less frequently, acquired esophageal strictures have been reported in human patients receiving alendronate,^37^, ^38^ but have not been reported in cats, possibly because of the lesser importance of gastroesophageal reflux disease in cats than in humans. In humans, it is advised to take alendronate in the morning with a full glass of water on an empty stomach in an upright position, without chewing or crushing tablets, and without lying down for 30 minutes after intake to decrease the risk of adverse reactions. Thus, it seems reasonable to recommend administration of water with alendronate in cats to prevent retention within the esophagus.

One cat in our cohort developed severe hypophosphatemia, prompting treatment discontinuation. Hypocalcemia, hypomagnesemia and hypophosphatemia are well‐known adverse effects of bisphosphonate treatment in humans, but hypophosphatemia is usually mild and self‐limiting once treatment is discontinued.^39^, ^40^ Interestingly, and similar to the cat in our cohort, a recent paper reported a woman who developed severe hypophosphatemia (0.62 mg/dL; reference interval, 2.4‐4.4 mg/L) and who did not have any associated clinical signs (eg, neuromuscular symptoms, muscle or bone pain, rhabdomyolysis, hemolytic anemia, cardiac arrhythmias). Thus, frequent evaluations of serum phosphate and other electrolyte concentrations should be performed in cats receiving alendronate, because severe life‐threatening phosphate depletion can be observed even in the absence of clinical signs.

The use of alendronate also has been associated with adverse osseous effects in humans, including increased risk of long bone fractures^41^ and osteonecrosis of the jaw.^19^ Skeletal complications are thought to occur secondary to alendronate‐induced decreased bone remodeling resulting in increased bone brittleness^42^ and to extensive osteoclast death within regions of high bone turnover (eg, maxillary bone).^43^ Administration of treatment over a long duration of time has been associated with increased risk of adverse osseous effects, probably because of the extremely long half‐life of bisphosphonates (exceeding 10 years in humans and 3 years in dogs).^44^ Both of these complications have been reported, although rarely, in cats treated with alendronate,^19^, ^20^ such that some authors recommend biannual radiographs of the tibia and the femur in treated cats to detect such adverse effects.^1^ None of the cats in our study underwent follow‐up radiographs, and it is possible that bone toxicity was underdiagnosed in our cohort.

The question of renal safety in people treated with bisphosphonates has been raised, because postinfusion increases in serum creatinine concentration have been reported in patients receiving IV ibandronate and zoledronic acid. However, clinical trial results have shown that even in patients with preexisting renal impairment, bisphosphonate treatment was not associated with a long‐term decline in renal function.^45^ In our cohort, occasional (but sometimes marked) increases in serum creatinine concentration were observed, but they were more likely attributable to spontaneous progression of chronic kidney disease or to episodes of ureteral obstructions than to alendronate toxicity. The median variation in serum creatinine concentration was not different between the 2 groups.

Our study had some limitations. First, age at inclusion and iCa_*t*0_ were not similar between the 2 groups, which may have biased univariate analyses by confounding bias. Second, inclusion of a small number of cats resulted in a lack of statistical power, and we hope our findings encourage additional studies with higher numbers of animals to provide evidence of a possible effect of alendronate treatment in persistent hypercalcemia. Third, because of the retrospective nature of our study, the number and timing of reevaluations as well as the total duration of follow‐up and type of food changes were not standardized between groups, which might have resulted in confounding bias in the interpretation of the results. Finally, finding an indicator that is relevant for documenting changes in a biochemical variable such as iCa over time was not easy. Indeed, most indicators provide information either on the magnitude of the decrease in iCa or its durability over time, but none simultaneously provides both pieces of information. This difficulty is common in both the veterinary and human medical literature. We believe a better way to address these concerns would be to use a randomized prospective study with preestablished follow‐up timepoints. Based on the hypothesis that 50% of individuals treated with alendronate and 20% of individuals receiving placebo would experience a 15% decrease in iCa, 70 individuals would be required to detect a significant difference (at the 5% level) in alendronate superiority in a randomized placebo‐controlled trial, with 80% statistical power.

## 5 CONCLUSION

Ours is the first study to report a cohort of cats with persistent hypercalcemia that were treated with alendronate for an extended follow‐up period (>6 months). Too little evidence has been gathered to strongly recommend treatment with alendronate rather than other therapeutic modalities in this context. Alendronate was, however, well tolerated in our cohort, and treatment with alendronate still might be considered in the management of cats with persistent hypercalcemia, in association with nutritional management. Additional controlled studies are needed to assess the efficacy of alendronate. Close monitoring is advised for the early detection of treatment‐associated complications, because related clinical signs could be absent.

## CONFLICT OF INTEREST DECLARATION

Authors declare no conflict of interest.

## OFF‐LABEL ANTIMICROBIAL DECLARATION

Authors declare no off‐label use of antimicrobials.

## INSTITUTIONAL ANIMAL CARE AND USE COMMITTEE (IACUC) OR OTHER APPROVAL DECLARATION

Authors declare no IACUC or other approval was needed.

## HUMAN ETHICS APPROVAL DECLARATION

Authors declare human ethics approval was not needed for this study.
